# Development and performance of a sleep estimation algorithm using a single accelerometer placed on the thigh: an evaluation against polysomnography

**DOI:** 10.1111/jsr.13725

**Published:** 2022-09-27

**Authors:** Peter J. Johansson, Patrick Crowley, John Axelsson, Karl Franklin, Anne Helene Garde, Pasan Hettiarachchi, Andreas Holtermann, Göran Kecklund, Eva Lindberg, Mirjam Ljunggren, Emmanuel Stamatakis, Jenny Theorell Haglöw, Magnus Svartengren

**Affiliations:** ^1^ Department of Medical Sciences, Occupational and Environmental Medicine Uppsala University, Uppsala University Hospital Uppsala Sweden; ^2^ The National Research Centre for the Working Environment Copenhagen Denmark; ^3^ Department of Psychology, Department of Clinical Neuroscience Stress Research Institute, Karolinska Institutet, Stockholm University Stockholm Sweden; ^4^ Department of Surgical and Perioperative Sciences, Surgery Umeå University Umeå Sweden; ^5^ Charles Perkins Centre, Faculty of Medicine and Health, School of Health Sciences University of Sydney Sydney Australia

**Keywords:** actigraphy, activity tracker; wearables, physical activity, sedentary behaviour

## Abstract

Accelerometers placed on the thigh provide accurate measures of daily physical activity types, postures and sedentary behaviours, over 24 h and across consecutive days. However, the ability to estimate sleep duration or quality from thigh‐worn accelerometers is uncertain and has not been evaluated in comparison with the ‘gold‐standard’ measurement of sleep polysomnography. This study aimed to develop an algorithm for sleep estimation using the raw data from a thigh‐worn accelerometer and to evaluate it in comparison with polysomnography. The algorithm was developed and optimised on a dataset consisting of 23 single‐night polysomnography recordings, collected in a laboratory, from 15 asymptomatic adults. This optimised algorithm was then applied to a separate evaluation dataset, in which, 71 adult males (mean [SD] age 57 [11] years, height 181 [6] cm, weight 82 [13] kg) wore ambulatory polysomnography equipment and a thigh‐worn accelerometer, simultaneously, whilst sleeping at home. Compared with polysomnography, the algorithm had a sensitivity of 0.84 and a specificity of 0.55 when estimating sleep periods. Sleep intervals were underestimated by 21 min (130 min, Limits of Agreement Range [LoAR]). Total sleep time was underestimated by 32 min (233 min LoAR). Our results evaluate the performance of a new algorithm for estimating sleep and outline the limitations. Based on these results, we conclude that a single device can provide estimates of the sleep interval and total sleep time with sufficient accuracy for the measurement of daily physical activity, sedentary behaviour, and sleep, on a group level in free‐living settings.

## INTRODUCTION

Time spent asleep, in different physical activities, sedentary behaviours and postures (‘physical behaviour’) have important implications for health. These physical behaviours are interrelated over the 24‐h cycle. To date, most of our knowledge about the interplay between sleep, physical activities, sedentary behaviours, and health comes from self‐reported data (i.e., data reported by the participant themselves; Bull et al., 2020). Self‐report data on physical activity, sedentary behaviour, and sleep are influenced by bias, which can lead to inaccurate estimates of the duration (Cespedes et al., 2016; Ekblom et al., 2015; Shephard, 2003; Troiano et al., 2020).

Body‐worn accelerometers are emerging as a more objective alternative to self‐reported data. In particular, the use of a single accelerometer placed on the thigh is increasing, as demonstrated by their adoption in large cohort studies and consortiums globally (Stamatakis et al., 2020, Stevens et al., 2020). A single thigh‐worn accelerometer can provide accurate estimates of a wide range of daily physical behaviours from sitting, standing, and walking (Edwardson et al., 2016; Skotte et al., 2014; Stemland et al., 2015; Stevens et al., 2020) to stair climbing, running, cycling (Migueles et al., 2017; Skotte et al., 2014; Stevens et al., 2020), and lying down (Hettiarachchi et al., 2021; Lyden et al., 2016). More objective measures of a wide range of activities are increasing as recognition of the inherent interdependence between daily physical behaviours across 24 h grows (Troiano et al., 2020). Interdependence demands that we measure a range of physical behaviours across 24 h, including physical activity, sedentary behaviour, and sleep, so that we can understand how they interact and relate to health. There are established algorithms for deriving physical activity and sedentary behaviour from thigh‐worn accelerometers, which have been evaluated against the ‘gold standard’ (Edwardson et al., 2016; Skotte et al., 2014; Stemland et al., 2015; Stevens et al., 2020; Migueles et al., 2017; Hettiarachchi et al., 2021; Lyden et al., 2016). However, we lack algorithms for deriving sleep duration, or other dimensions of sleep that could indicate sleep quality from thigh‐worn accelerometers, which have also been evaluated against the gold standard of polysomnography (PSG). To the best of our knowledge, there are just two published non‐proprietary algorithms for deriving ‘waking time’ and a single proprietary algorithm that estimates ‘bedtime’ using the data from thigh‐worn accelerometers (Carlson et al., 2021; van der Berg et al., 2016; Winkler et al., 2016). None of these algorithms have been evaluated against the gold standard (Carlson et al., 2021; Inan‐Eroglu et al., 2021; van der Berg et al., 2016; Winkler et al., 2016). Similarly, although numerous studies exist evaluating the use of hip‐ and wrist‐worn accelerometers to measure several dimensions of sleep (Conley et al., 2019), we are not aware of any evaluations that have used thigh‐worn accelerometers to measure anything else than ‘bedtime’ or ‘waking time’.

Our primary aim was to develop a non‐proprietary algorithm for the estimation of sleep duration, derived using the raw data from a tri‐axial thigh‐worn accelerometer, and to evaluate this algorithm against PSG. Our secondary aim was to evaluate the performance of the algorithm when used to estimate other sleep quality variables such as: sleep latency, wake after sleep onset (WASO), sleep efficiency, and awakening index.

## SUBJECTS AND METHODS

The algorithm was developed using a two‐step process. In step one, the algorithm was optimised to maximise the sensitivity and specificity of sleep estimation in comparison with PSG recordings collected in a sleep laboratory (see below and Table 1). We refer to this dataset as the *optimisation dataset*. In step two, the optimised algorithm was tested on a new dataset, consisting of ambulatory PSG recordings collected from a new sample of participants, at their homes (see below and Table 1). We refer to this dataset as the *evaluation dataset*. Step two was performed because the performance of the algorithm may differ from laboratory conditions to ambulatory/free‐living conditions. As our algorithm is intended for use in free‐living settings, the results section will focus on the performance of the algorithm when applied to the evaluation dataset.

**TABLE 1:** Characteristics of the separate samples of participants in the optimisation and ambulatory validation dataset

	Optimisation dataset (*N* = 15; 8 females, 7 male)				Validation dataset (*N* = 71; all male)			
	Mean	SD	Median	Range	Mean	SD	Median	Range
Age, years	28	5.3	28	22–38	57	11.2	60	34–73
Weight, kg	65	11.1	67	49–82	82	12.6	81	59–113
Height, cm	169	10.1	167	158–190	181	5.5	181	166–192
Body mass index, kg/m^2^	23	2.6	23	19–27	25	3.9	24	18–35

Informed consent was provided by all participants prior to inclusion, in accordance with the Helsinki Declaration. For the optimisation dataset, data collection was approved by the Regional Ethics Review Board in Stockholm (identification [ID] number: 2016/193), with additional approval for adding extra participants and accelerometers on the thigh (ID number: 2018/2196‐32) and by the Scientific Ethics Committee for the capital region, Denmark (ID number: 18005389). For the evaluation dataset, data collection was approved by the local Ethics Committee in Uppsala, Sweden (ID number: 2016/029), with an additional approval for the addition of accelerometers on the thigh (ID number: 2016/029/1).

### Optimisation dataset

A total of 23 overnight PSG recordings were obtained from 15 asymptomatic adults between the ages of 22 and 38 years (Table 1) at the Stress Research Institute, Stockholm University, Sweden. Participants were recruited through a university website and offered a small monetary compensation for participation and a complimentary breakfast. Data were collected during late autumn in 2018. Exclusion criteria included any previously diagnosed sleep disorder, self‐reported poor sleep quality or the use of medication for any sleep‐related problems. Moreover, participants were excluded if they reported finding it difficult to sleep in other places than one's own bed or having insomnia symptoms such as difficulties falling asleep and waking up often without being able to go back to sleep. Further exclusion also included participants who reported heavy snoring, having current physical or mental health problem, heavy consumption of alcohol or drugs, shift work, having travelled more than one time zone in the last 3 weeks, pregnancy, fever, or allergy to adhesive bandages. All participants in the optimisation dataset, underwent a preliminary overnight recording to allow for habituation to the sleep laboratory and study protocol. This recording was also used for screening of sleep disorders and restless leg syndrome. Participants were monitored for at least an additional 1 night in a controlled laboratory environment. A further eight participants completed 2 non‐consecutive nights of PSG recordings. To increase the data available for algorithm optimisation, all recordings were included. For the full PSG assessment (TEMEC Technologies, VitaPort 3, Heerlen, the Netherlands), PSG electrodes were placed according to the American Association of Sleep Medicine (AASM) guidelines (Berry et al., 2015) and included two electroencephalography (EEG) leads (C3‐A2 and C4‐A1), two electro‐occulogram (EOG) leads, and submental electromyogram (EMG). In addition, a single accelerometer (Axivity AX3, Axivity Ltd, Newcastle Upon Tyne, UK) was attached with adhesive tape, on the participant's thigh, midway between the patellar ligament and the anterior superior iliac spine. Acceleration data were sampled at a frequency of 100 Hz, with a range ± 8 *g*. To ensure synchronisation between devices, a ‘synchronisation event’ at the start and end of each registration was included. In short, when participants were equipped with all devices and recording active, they were asked to step out of bed and stand still in an upright position for 15 s. Then, they were asked to bite their teeth together three times, perform a single hop, and bite their teeth together a further three times, before again standing still for 10–15 s. This procedure provided a clearly identifiable event in the signals of the various devices, providing a reference point for time synchronisation.

### Evaluation dataset

The evaluation dataset consisted of a single night of ambulatory PSG registration from 71 males recruited from the ‘Men in Uppsala; a Study of sleep, Apnea and Cardiometabolic Health’ (MUSTACHE) study (Table 1). The MUSTACHE study is a population study initiated in 2016 and aimed at reaching 400 male participants within the age range of 35–65 years. The 71 males in the present study were recruited as a convenience sample from the last round of recruitment in the MUSTACHE study. Participants who were not expected to manage to carry out the ambulatory recordings due to self‐reported severe somatic or psychiatric disease were excluded. All participants wore ambulatory PSG equipment (Embla Flaga Inc., Reykjavik, Iceland). The PSG recording included EEG (C3‐A2, C4‐A1), EOG and submental EMG. Additional sensors used were electrocardiograms (V5), airflow with a three‐port orinasal thermistor and a nasal flow pressure sensor, respiratory effort from piezo‐electric belts (Resp‐Ez, EPM Systems, Midlothian, VA, USA), bilateral anterior tibialis muscles, finger pulse oximetry (Embla A10 flex sensor), a piezo vibration sensor for snoring, and a body position sensor. In addition, a single accelerometer (Axivity AX3, Axivity Ltd) was attached with adhesive tape, on the participant's thigh, midway between the patellar ligament and the anterior superior iliac spine. Acceleration data were sampled at a frequency of 25 Hz, with a range of ±8 *g*. Participants were required to attend an afternoon appointment on the first day, where trained personnel ensured correct sensor placement. Thereafter, participants returned home and wore the sensors continuously until the following morning. PSG recordings began once the participants went to bed and stopped when they awoke the following morning. Each accelerometer and PSG recording was synchronised and then visually inspected to ensure synchronisation was correct. Data collection was carried out between July 2018 and May 2019.

### The sleep algorithm

**FIGURE 1:**
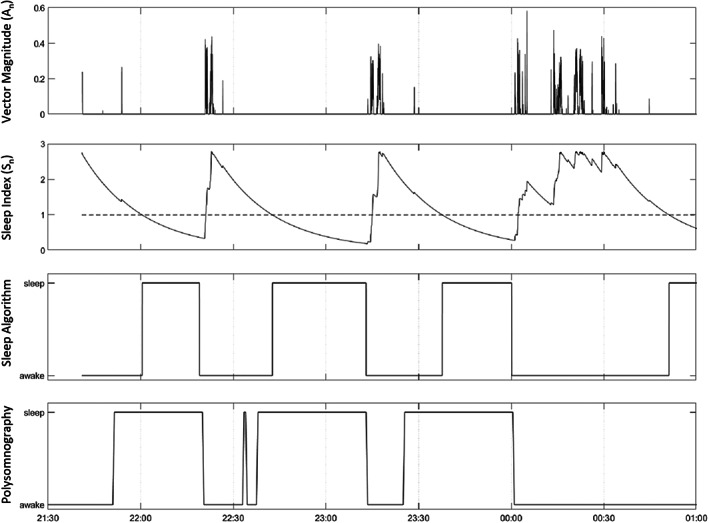
An example portraying (from top to bottom) vector magnitude of thigh accelerations, the sleep index (Sn), sleep and awake according to the sleep algorithm, and sleep and awake according to polysomnography

A simple algorithm was developed to estimate sleep from raw accelerometer data, based on the algorithm of Cole–Kripke (Cole et al., 1992). Wake and sleep thresholds were set for each second of lying periods >15 min, based on a constantly changing variable, called the ‘sleep index’ ($S_{n})$. A sleep index >1 was considered as ‘awake’ and a sleep index <1 was considered as ‘asleep’ (see Formula 1). Thus, thigh movement would increase the value of the sleep index, and time without thigh movement would decrease the value of the sleep index (Figure 1).

Raw accelerometer data were re‐sampled to 30 Hz. Data were then band‐pass filtered 0.5–10 Hz, whilst further background noise was removed from the signal using a cut‐off value of 0.02 *g*. Then, the sleep index was calculated using the following formula:

(1) $$S_{n}=\exp\left(\frac{-1}{\tau }\right)*S_{n-1}+k*A_{n},$$

Where

‘*A*_*n*_’ is the mean band‐pass‐filtered vector magnitude in *n* ^th^ second.

*‘τ’* is the time constant.

*‘k’* is the gain parameter.

**FIGURE 2:**
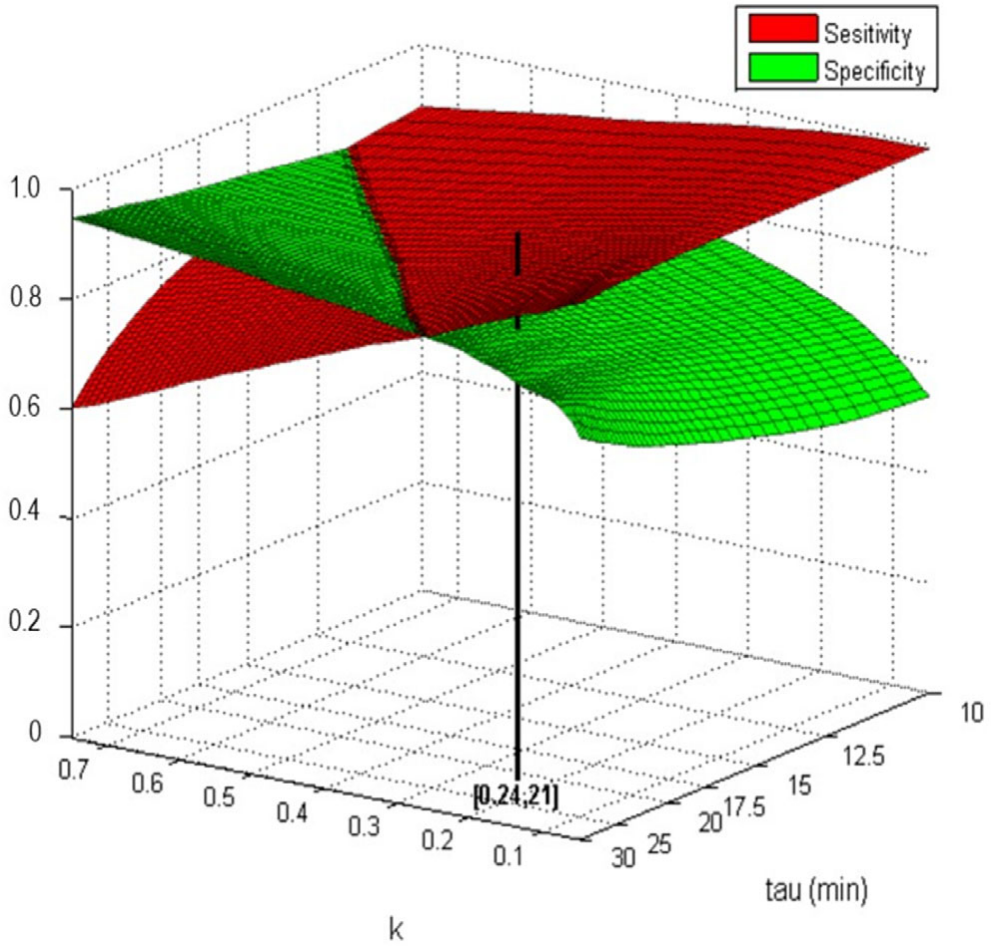
Sensitivity and specificity of the algorithm to identify sleep time *in the optimisation dataset* for different values of the constants *τ and k*

Both τ and k are held constant and were optimised through iterative comparison between the algorithm output and PSG recordings, as described in paragraph 2.5 (Figure 2). An upper limit of exp(1) = 2.71 was set for $S_{n}$, meaning that when $A_{n}$ = 0, the value of $S_{n}$ decreases exponentially in line with the time constant τ (i.e., sleep is only defined after *τ*‐seconds, if no further movements are detected) (Figure 1). Sleep or wake‐state bouts that lasted <10 s were removed using a median filter. Furthermore, in order to account for the time taken for the sleep index $\left(S_{n}\right)$ to rise above the movement detection threshold during awakening, each awakening was considered to occur 2 min prior.

### Data analysis

The PSG recordings were scored in 30‐s epochs according to standard criteria to detect sleep (Ancoli‐Israel et al., 2007; Rechtschaffen & Kales, 1968). This was performed separately by trained specialists for both the optimisation and evaluation datasets.

The output from the accelerometer algorithm was down‐sampled with mode‐filtering in order to fit the same 30‐s epoch lengths as PSG. Then the thigh accelerometer data and PSG recordings were synchronised and manually inspected to assure that the time synchronisation was correct. Epoch‐by‐epoch comparisons were then made to calculate the sensitivity, specificity, and accuracy of the sleep algorithm to estimate sleep with respect to PSG detection, according to the following formulas:

$$\text{Sensitivity}=\frac{\mathrm{TP}}{\mathrm{TP}+\mathrm{FN}}$$

$$\text{Specificit}y=\frac{\mathrm{TN}}{\mathrm{TN}+\mathrm{FP}}$$

$$\text{Accuracy}=\frac{\mathrm{TP}+\mathrm{TN}}{\mathrm{TP}+\mathrm{TN}+\mathrm{FP}+\mathrm{FN}}$$

where

TP (true positive) = the number of epochs flagged as ‘asleep’ both from the accelerometer and PSG.

FP (false positive) = the number of epochs flagged as ‘asleep’ by the accelerometer but flagged as wake according to PSG.

TN (true negative) = the number of epochs flagged as ‘awake’ both from the accelerometer and PSG.

FN (false negative) = the number of epochs flagged as ‘awake’ by the accelerometer but flagged as sleep by the PSG.

### Optimisation

To optimise the constants τ and k in Formula 1, the sum of sensitivity and specificity statistics over all recordings were evaluated iteratively for different values of τ and k in the optimisation dataset. A constant value τ = 18.5 min and *k* = 0.19 proved optimal, resulting in a sensitivity of 0.90 and specificity of 0.85 derived from the optimisation dataset (Figure 2).

### Performance evaluation

To assess the performance of the optimised algorithm, comparison between sleep defined by ambulatory PSG recordings and sleep defined by the sleep algorithm was performed in the evaluation dataset.

The mean and standard deviation (SD) of sensitivity, specificity and accuracy was calculated for all participants. In addition, the following variables were derived from both the PSG recordings and the sleep algorithm according to Ibáñez et al. (2018): *sleep interval*, is defined as the time between the onset of the first sleep period and the last awakening; *total sleep time*, is defined as the total amount of time the participants slept between the start of the PSG recording until the last awakening, identified by PSG; *sleep latency*, is defined as the time from the start of the PSG recording until the time the participant fell into a stage of sleep for the first time*; WASO*, is defined as the total time awake after the first sleep onset until the last awakening; *sleep efficiency*, is defined as the percentage of recorded time asleep until the last awakening; *awakening index*, is defined as the number of awakenings >10 s per hour.

**FIGURE 3:**
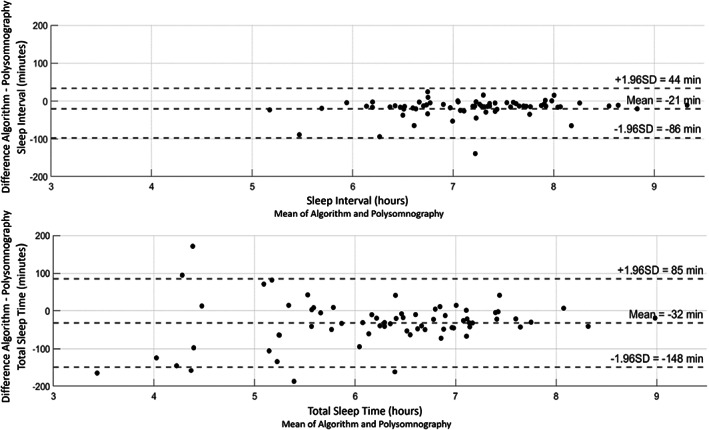
Bland–Altman plots showing the individual differences between the sleep algorithm and polysomnography estimated sleep interval (upper) and total sleep time (lower), in minutes. Each participant is represented as a dot. The upper and lower dotted line represent the 95% limits of agreement and the middle‐dotted line represents the mean differences

The difference between the sleep variables derived from PSG recordings and that of sleep variables from the algorithm was calculated. The upper and lower 95% limits of agreement (LoA) of the sleep interval and total sleep time were also calculated by taking the mean differences ±1.96 × SD of the differences (Bland & Altman, 1999) and presented in Bland–Altman plots (Figure 3 and Table 3). The range between the upper and lower LoA (LoA Range [LoAR]) was used as a measure of precision for comparison with earlier studies. Correlation between the aforementioned variables between the sleep algorithm and PSG was calculated with Pearson correlation.

In order to assess if outliers (i.e., those who had very short or very long sleep duration relative to the norm) affected results, a sensitivity analysis was performed excluding cases that had slept shorter than or longer than 2 SDs from the mean sleep time, according to the PSG recordings. All analysis was performed with Matlab 2020b Windows version and Rstudio 2021.09.1.

## RESULTS

Summary of sleep parameters derived from the PSG and the sleep algorithm in the evaluation dataset are presented in Table 2.

**TABLE 2:** Sleep parameters from the polysomnography recordings and thigh accelerometer sleep algorithm in the validation dataset, *N* = 71

	Polysomnography			Sleep algorithm		
	Mean	SD	Range	Mean	SD	Range
Sleep interval, min	441	45	322–565	420	53	252–554
Total sleep time, min	392	67	177–549	360	75	124–530
Sleep latency, min	10	13	0.5–107	33	33	19–222
Wake after sleep onset, min	50	49	0–223	60	48	0–243
Sleep efficiency, %	86	12	41–99	79	13	31–93
Awakening index, *n*/h	1.6	0.7	0.1–4.0	0.5	0.3	0.0–1.4

The sleep algorithm detected sleep with a mean (SD; range) sensitivity of 0.84 (0.12; 0.34–0.84), specificity of 0.55 (0.25; 0.07–1.00) and accuracy of 0.80 (0.10; 0.45–0.95) when applied to the evaluation dataset. The sleep algorithm on average underestimated the sleep interval by 21 min (LoAR = 130) and total sleep time by 32 min (LoAR = 233) (Table 3, Figure 3). The highest correlation between PSG and the sleep algorithm was observed for sleep interval and total sleep time (Table 3).

**TABLE 3:** Bland–Altman statistics, mean differences (bias), lower and upper limits of agreement (LoA) and correlation between polysomnography‐ and accelerometer‐derived sleep parameters, *N* = 71

	Bias	95% CI	Lower LoA	95% CI	Upper LoA	95% CI	Pearson correlation, *r*	95% CI
Sleep interval (min)	−21	−29, −13	−86	−99, −72	44	31, 57	0.78	0.68, 0.86
Total sleep time (min)	−32	−46, −18	−148	−173, −124	85	61, 109	0.66	0.5, 0.77
Sleep latency (min)	23	15, 30	−38	−51, −26	83	71, 96	0.37	0.16, 0.56
Wake after sleep onset (min)	10	−1.1, 22	−84	−103, −64	104	85, 124	0.52	0.33, 0.67
Sleep efficiency (%)	−7.2	−10, 4	−34	−40, −29	20	14.1, 25	0.42	0.21, 0.6
Awakening index (*n*/h)	−1.1	−1.2, −0.9	−2.3	−2.5, −2	0.1	−0.2, 0.3	0.49	0.28, 0.65

Abbreviation: CI, confidence interval.

Using threshold values of ±2 SDs from the mean sleep time according to PSG recordings, three outliers were identified. A subsequent sensitivity analyses excluding these outliers indicated that the correlations between accelerometer measured, and PSG measured total sleep time increased to *r* = 0.70 (0.55–0.80), WASO increased to *r* = 0.57 (0.38–0.71) and sleep efficiency increased to *r* = 0.49 (0.29–0.65). Bias of total sleep time increased with 5–37 min but LoAR decreased with 25–208 min. Estimates of the sleep interval, sleep efficiency, and the awakening index did not alter.

## DISCUSSION

We developed and optimised an algorithm to estimate sleep using a thigh‐worn accelerometer using laboratory PSG recordings, and then, validated the performance of this algorithm against ambulatory PSG on a separate sample of participants measured in free‐living conditions. The algorithm demonstrated good sensitivity (0.84) and accuracy (0.80) and moderate specificity (0.55) when compared with ambulatory PSG recordings. This performance is comparable to the findings of previous research attempting to estimate sleep using wrist‐worn accelerometers amongst healthy adults, where a mean sensitivity, accuracy, and specificity of 0.89, 0.88, and 0.53, respectively, has been reported (Conley et al., 2019).

**FIGURE 4:**
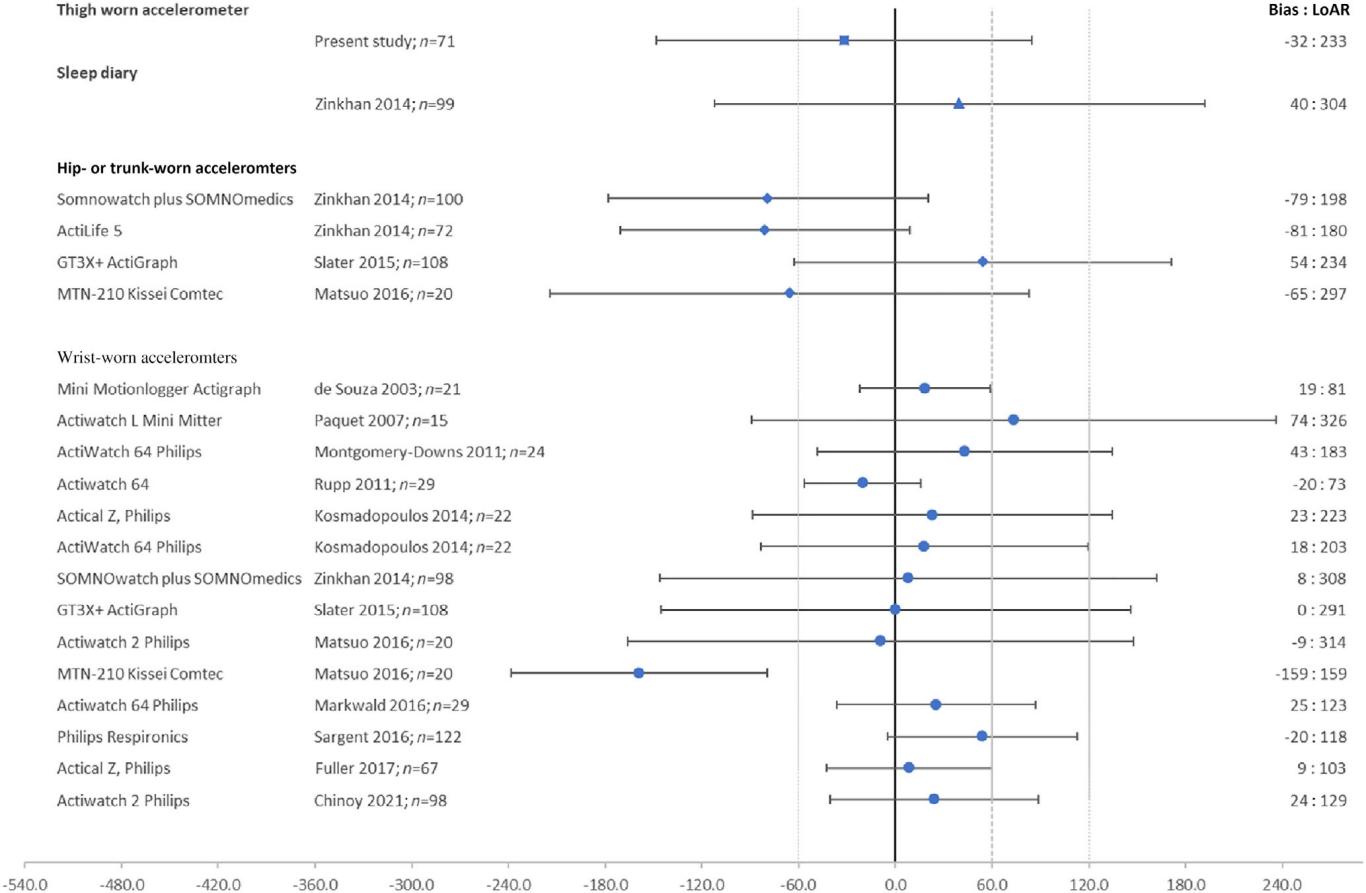
Performance of diary, and different accelerometer placements in measuring total sleep time (TST) compared to polysomnography. Differences in minutes, bias and 95% limits of agreement ±2 SD (limits of agreement range), (*n* = number of nights). Only studies with healthy adult populations and studies that have presented the mean differences (bias) and SD of differences of TST between the two methods are presented

The variable that corresponded best between the algorithm and PSG was sleep interval, which was underestimated by 21 min (LoAR 130 min), equating to 5% of the total sleep interval registered by PSG. Total sleep time was underestimated by 32 min (LoAR 233 min) on average, equating to 7% of total sleep time registered by ambulatory PSG. This estimation appears to be somewhat better than sleep diary registrations of total sleep time (Zinkhan et al., 2014) and is in line with the performance of hip‐, trunk‐, (Matsuo et al., 2016; Slater et al., 2015; Zinkhan et al., 2014) and wrist‐worn accelerometer placements, even though the results from earlier validation studies of accelerometers are heterogeneous in relation to the estimation of total sleep time (Chinoy et al., 2021; Conley et al., 2019; de Souza et al., 2003; Fuller et al., 2017; Kosmadopoulos et al., 2014; Markwald et al., 2016; Matsuo et al., 2016; Montgomery‐Downs et al., 2012; Paquet et al., 2007; Rupp & Balkin, 2011; Sargent et al., 2016; Slater et al., 2015; Zinkhan et al., 2014) (Figure 4).

Our algorithm underestimated total sleep time by 32 min, whereas the previous trend for wrist‐worn accelerometer estimates of sleep has been towards an overestimation by ~11 min (Conley et al., 2019). An explanation for this discrepancy may lie in the algorithm design, where each awakening was considered to occur 2 min prior to the time indicated by sleep index ($S_{n})$. Another reason for underestimation could be that the algorithm always takes at least 18.5 min, due to the time constant τ, to detect sleep‐onset from a full awake status, even though in reality people can wake‐up and go back to sleep very quickly. These parameters were selected to optimise specificity but may have inadvertently contribute to the underestimation observed. Alternatively, the observed underestimation may indicate that thigh movement during sleep is more prevalent than the movement of other body parts, although further research is needed to confirm this.

Relatively low precision (high LoAR) was observed for all variables indicating that the algorithm is mainly suited to studies where *groups* of individuals are evaluated. We also observed poor correlations between PSG‐ and algorithm‐derived sleep quality variables, including sleep latency; sleep efficiency; WASO; and awakening index. This suggests that the algorithm is perhaps less suited to the measurement of sleep quality. However, the performance is still comparable with many observations using wrist‐worn accelerometer (Conley et al., 2019). The poor correlation across these variables is likely a result of the fact that the sleep state is a multifactorial physiological process. Thus, short awakenings or sleep episodes may not be related to any particular changes in thigh movement. Therefore, if the estimation of these sleep quality variables with high precision is of priority, alternative measurement methods should be considered.

When interpreting the results and comparisons above, the following three points are important to consider. Firstly, the comparison of sensitivity and specificity statistics across studies should not be considered as definitive, because differences in the total recorded sleep time, and prevalence of sleep between the studies can affect the statistic. Secondly, the comparison of findings using laboratory‐based PSG with those using ambulatory PSG should not be considered as a one‐to‐one comparison, because ambulatory PSG is likely to contain much more natural variation than laboratory‐based PSG. Thirdly, as is evident from Figure 3, short sleepers (<6 h) appear to have introduced greater disagreement between PSG and accelerometer estimations than those who slept longer (>6 h). This was also shown in the sensitivity analysis where the precision slightly increased (i.e., LoAR decreased) for total sleep time when very short sleepers were excluded from the analysis. Therefore, it is important to consider that the performance of the algorithm might vary depending on the population measured. The precision will most likely be lower amongst groups of individuals with sleep durations that are considerably shorter than the norm. It has also been shown, in other studies, that accelerometer estimates of sleep are better when applied to healthy populations than to populations with chronic disorders (Conley et al., 2019). For example, the performance of our algorithm may have been affected by the fact that participants with sleep disorders were excluded in the optimisation dataset but not in the evaluation dataset. In theory disorders like periodic limb movements and restless legs may affect the sleep estimations that are based on thigh movements. Finally, the performance may also have been affected by the fact that there were two different scorers that performed the sleep scoring in the optimisation and evaluation datasets.

Our findings are strengthened by comparison against the ‘gold standard’ for sleep measurement and the evaluation of the algorithm using a separate dataset from the dataset it was developed on. Another strength is that this evaluation was made using data collected in free‐living settings. However, PSG data were only recorded during the night, meaning that our results can only tell us how well the algorithm performs to estimate sleep during a given time‐window and not over 24 h. This is a problem frequently encountered when comparing sleep algorithms with PSG recordings. A further consideration is that the evaluation dataset consisted entirely of males between the ages of 34 to 73 years. As people age the pattern of sleep behaviours and prevalence of sleep disorders increases as life and health circumstances change. Therefore, the dissimilarity between the datasets may account for some of the decrease in performance of the algorithm from the optimisation dataset to the evaluation dataset.

The development and evaluation of this algorithm has a number of important practical implications. The first is that a thigh‐worn accelerometer performs almost as well as the traditional wrist‐worn accelerometers in the estimation of total sleep time. This has important implications in research integrating sleep, physical activity, and sedentary behaviour. Wrist‐worn accelerometers are a feasible tool for sleep measurement (Conley et al., 2019), but there are limitations when measuring other daily physical behaviours. Neither wrist‐ nor hip‐worn accelerometers can delineate physical activity and posture types, and moreover, arm movements can introduce measurement errors of total physical activity estimation when wrist‐worn accelerometers are used (Migueles et al., 2017). Thigh‐worn accelerometers have the advantage of being able to distinguish different physical activities and postures (like sitting, lying, standing, walking, running, stair‐walking, bicycling [Hettiarachchi et al., 2021; Lyden et al., 2016; Skotte et al., 2014; Stemland et al., 2015]), and now, can also be considered as a valid option for future device‐based sleep measurement. Another implication is that the algorithm is non‐proprietary, transparent, and not dependent on the brand of accelerometer. Such non‐proprietary algorithms are necessary to fulfil the needs of researchers in the field of physical activity, sedentary behaviour, and sleep measurement, and to achieve scientific goals of emerging consortia, such as, the Prospective Physical Activity, Sedentary behaviour and Sleep (ProPASS) consortium (Stamatakis et al., 2020).

## CONCLUSION

This study proposes a simple, transparent approach for estimating sleep time from thigh‐worn accelerometers. Our findings show that a thigh‐worn accelerometer was sensitive to sleep periods, but not sensitive to periods where the participant awoke during the night without detected thigh movements. Thus, our algorithm is less appropriate for the measurement of sleep quality variables.

Our method performs almost as well as the traditional wrist‐worn accelerometers (actigraphy) for the estimation of total sleep time. This demonstrates that there is now a feasible method for measuring sleep, physical activity, sedentary behaviour, and postures, on a group level, with just a single accelerometer.

## AUTHOR CONTRIBUTIONS

Conceptualisation: Magnus Svartengren, Peter J. Johansson, Patrick Crowley, Anne Helene Garde, and Andreas Holtermann; Methodology: Magnus Svartengren, Peter J. Johansson, Patrick Crowley, Andreas Holtermann, Anne Helene Garde, and Pasan Hettiarachchi; Software: Pasan Hettiarachchi; Evaluation: Pasan Hettiarachchi, and Peter J. Johansson; Formal Analysis: Magnus Svartengren, Peter J. Johansson, Patrick Crowley, and Andreas Holtermann; Investigation: Eva Lindberg and Göran Kecklund; Resources: Eva Lindberg, John Axelsson, and Göran Kecklund; Data Curation: Peter J. Johansson, Pasan Hettiarachchi, Eva Lindberg, Patrick Crowley, Jenny Theorell Haglöw, and Karl Franklin; Writing – Original Draft Preparation: Peter J. Johansson and Patrick Crowley; Writing – Review and Editing:, Peter J. Johansson, Patrick Crowley, John Axelsson, Karl Franklin, Anne Helene Garde, Pasan Hettiarachchi, Andreas Holtermann, Göran Kecklund, Eva Lindberg, Mirjam Ljunggren, Emmanuel Stamatakis, Jenny Theorell Haglöw, and Magnus Svartengren; Visualisation: Pasan Hettiarachchi and Peter J. Johansson; Project Administration, Peter J. Johansson. Funding Acquisition: Peter J. Johansson, Andreas Holtermann, Emmanuel Stamatakis, Eva Lindberg, John Axelsson, Magnus Svartengren, and Göran Kecklund.

## FUNDING INFORMATION

This research was partly financed by grants from: the Swedish state under the agreement between the Swedish government and the county councils, the ALF‐agreement (1040232); FORTE, Swedish Research Council for Health, Working Life and Welfare (2021–01561); The Danish Work Environment Research Fund (November 03 2017); National Health and Medical Research Council Investigator Grant, Leadership second level (APP1194510); British Heart Foundation, Special Grant (SP/F/20/150002); National Health and Medical Research Council Ideas Grant (APP1180812); Swedish Heart Lung Foundation (20160343); Funding from Stockholm Stress Center – a centre of excellence for research on work‐related stress and health Stress Research; AFA‐insurance (150159).

## CONFLICT OF INTEREST

The authors declare no conﬂict of interest.

## Data Availability

The data that support the findings of this study are available from the corresponding authors (Peter J. Johansson P.J. and Patrick Crowley), after agreement with the Principal Investigators of the data sources (John Axelsson, Göran Kecklund and Eva Lindberg) upon reasonable request as long as it does not compromise the privacy of research participants or any Swedish or European Union regulations.
